# Advancing pharmacogenetic testing in a tertiary hospital: a retrospective analysis after 10 years of activity

**DOI:** 10.3389/fphar.2023.1292416

**Published:** 2023-10-19

**Authors:** Stefan Stewart, Jose Manuel Dodero-Anillo, Javier Guijarro-Eguinoa, Pedro Arias, Arturo Gómez López De Las Huertas, Enrique Seco-Meseguer, Irene García-García, Elena Ramírez García, Carlos Rodríguez-Antolín, Antonio J. Carcas, Sonia Rodriguez-Novoa, Rocio Rosas-Alonso, Alberto M. Borobia

**Affiliations:** ^1^ Clinical Pharmacology Department, IdiPAZ, La Paz University Hospital, Madrid, Spain; ^2^ Clinical Pharmacology, Hospital Universitario Puerto Real, Cadiz, Spain; ^3^ Pharmacogenetics Laboratory, Genetics Department, La Paz University Hospital, Madrid, Spain; ^4^ Pharmacology Department, School of Medicine, Universidad Autónoma de Madrid, Madrid, Spain; ^5^ Experimental Therapies and Novel Biomarkers in Cancer, Hospital La Paz Institute for Health Research—IdiPAZ, Madrid, Spain; ^6^ Genetics of Metabolic Diseases Laboratory, Genetics Department, La Paz University Hospital, Madrid, Spain

**Keywords:** pharmacogenetic dosing, preemptive genotyping, real-world data, personalized medicine, retrospective analysis

## Abstract

The field of pharmacogenetics (PGx) holds great promise in advancing personalized medicine by adapting treatments based on individual genetic profiles. Despite its benefits, there are still economic, ethical and institutional barriers that hinder its implementation in our healthcare environment. A retrospective analysis approach of anonymized data sourced from electronic health records was performed, encompassing a diverse patient population and evaluating key parameters such as prescribing patterns and test results, to assess the impact of pharmacogenetic testing. A head-to-head comparison with previously published activity results within the same pharmacogenetic laboratory was also conducted to contrast the progress made after 10 years. The analysis revealed significant utilization of pharmacogenetic testing in daily clinical practice, with 1,145 pharmacogenetic tests performed over a 1-year period and showing a 35% growth rate increase over time. Of the 17 different medical departments that sought PGx tests, the Oncology department accounted for the highest number, representing 58.47% of all genotyped patients. A total of 1,000 PGx tests were requested for individuals susceptible to receive a dose modification based on genotype, and 76 individuals received a genotype-guided dose adjustment. This study presents a comprehensive descriptive analysis of real-world data obtained from a public tertiary hospital laboratory specialized in pharmacogenetic testing, and presents data that strongly endorse the integration of pharmacogenetic testing into everyday clinical practice.

## 1 Introduction

Within the rapidly advancing field of personalized medicine, pharmacogenetics has come to prominence as a growing discipline that examines how genetic variations influence an individual’s response to drugs. Variants in genes that encode drug-metabolizing enzymes, drug transporters, and drug targets can significantly impact the disposition and action of drugs, which ultimately leads to variability in response (Bielinski et al., 2014). This is particularly relevant in medications where there is a marginal difference between a therapeutic and a toxic dose (i.e., a narrow therapeutic window) (Tong et al., 2021). As a result, pharmacogenetic (PGx) testing performed either preemptively or *ad hoc* has become increasingly important as a means of individualizing treatment plans, enhancing treatment efficacy, and mitigating the occurrence of adverse events (García-García and Borobia, 2021).

Our understanding of the impact of genetic variations on drug response is continuously expanding, as advances in genotyping and sequencing technologies allow for further insight into the complex interactions between genetics, drugs, and individual patients (Bielinski et al., 2014). Numerous studies, including randomized controlled trials, have demonstrated the efficacy of personalized drug therapy based on PGx testing for specific drug-gene combinations (Tong et al., 2016). The Preemptive Pharmacogenomic Testing for Preventing Adverse Drug Reactions (PREPARE) study, conducted by the Ubiquitous Pharmacogenomics Consortium, and the more recent 12-gene PGx panel implementation study stand as particularly notable (Swen et al., 2023).

Despite encountering slow initial uptake and wavering levels of acceptance among physicians, the integration of PGx information into clinical practice has gained momentum due to mounting evidence (Borobia et al., 2018). The development of precise, easily accessible, and cost-effective molecular analysis techniques has also contributed to its increasing adoption. Consequently, regulatory bodies such as the US Food and Drug Administration (FDA) and the European Medicines Agency (EMA) have recognized the significance of PGx information and incorporated it into the drug label information making it readily available to both prescribers and patients (Borobia et al., 2018; Brunette et al., 2020; García et al., 2020; Díaz-Villamarín et al., 2022). In this context, clinical guidelines have been developed to aid prescribing clinicians, such as the ones by The Dutch Pharmacogenetics Working Group (DPWG) and the Clinical Pharmacogenetics Implementation Consortium (CPIC), which comprehensively evaluate the associations between over 100 gene-drug pairs (D’Andrea et al., 2015; Leusink et al., 2016; Díaz-Villamarín et al., 2022). More recently, the Spanish Society of Pharmacogenetics and Pharmacogenomics (SEFF) has developed its own clinical practice guidelines grounded in the national portfolio of services and the idiosyncrasies inherent to the Spanish National Health System (SNHS) (Sociedad Española de Farmacogenética y Farmacogenómica, 2023). These milestones coupled with the rise of precision medicine, have eventually led to the incorporation of pharmacogenetic biomarker testing into the services portfolio of many Health Services worldwide, including the SNHS (Borobia et al., 2018; García et al., 2020).

However, despite its potential benefits, there are still economic, ethical and institutional barriers that hinder its implementation beyond a limited number of tertiary hospitals in our healthcare environment (Borobia et al., 2018). One of the primary challenges faced is the lack of standardization within the field, which can lead to disparities in the interpretation of results, potentially precluding patients from receiving the benefits of personalized treatment plans or expose them to avoidable side effects (Weitzel et al., 2014).

In order to fully realize the potential of pharmacogenetics, clinical pharmacogenetic laboratories have a vital role to play in overcoming, not only the aforementioned challenges, but the current shortages of trained technical staff and insufficient expertise amongst healthcare personnel. This would not only improve patient outcomes by enhancing safety and effectiveness of medication use, but ultimately contribute to a more sustainable healthcare system (Borobia et al., 2018; Karamperis et al., 2021).

To assess the practical impact of pharmacogenetics in a real-world setting, we conducted a thorough descriptive analysis of annual data from the Clinical Pharmacogenetics Unit located within the La Paz University Hospital (LPUH), a tertiary public hospital equipped with over 1,300 beds that attends the population of northern Madrid. The LPUH Clinical Pharmacogenetics Unit was established in 2013 and its clinical protocols were developed by clinical pharmacologists and geneticists in collaboration with the clinical services that regularly request genetic tests (Borobia et al., 2018). Most of these protocols have well-defined guidelines for pharmacogenetic treatment recommendations. To further aid the petitioner in the optimization of dosing requirements or treatment selection (in the context of this study, we use the terms “petitioner” to refer to the individual ordering a pharmacogenetic test and “petitions” to denote the test orders), clinical and genetic information are assessed and integrated by a clinical pharmacologist who then emits individualised recommendations.

The primary objective of this study is to highlight the activity, challenges, and advancements achieved by our Clinical Pharmacogenetic Unit over a 1-year period. In addition, our study aimed to conduct a head-to-head comparison of real-world data from previously published activity results within the same tertiary Pharmacogenetic Unit, spanning two different time periods: period 1 (January 2014 and December 2016) and period 2 (August 2021 and September 2022). Ultimately, this study aims to contribute to the expanding evidence base that supports the integration of PGx testing into routine clinical practice.

## 2 Materials and methods

### 2.1 Study design

We conducted a retrospective, single-center study to assess the impact of PGx testing during routine clinical practice at the LPUH Clinical Pharmacogenetics Unit. Established in 2013, it operates as a multidisciplinary entity, integrating the Clinical Pharmacology Department and the Genetics Department, both of which hold ISO 9001:2015 certification. Both departments play an essential role within the Unit; the geneticist is responsible of the technical, analytical and genetic data interpretation, whereas the clinical pharmacologist provides expertise on possible drug interactions, integrates patient’s clinical status with the obtained phenotype, and emits individualized treatment plans to be prescribed by the petitioner. It has implemented treatment adjustment protocols for the following preemptively genotyped drug-gene pairs: thiopurines/*TPMT-NUDT15*, tacrolimus/*CYP3A5*, voriconazole/*CYP2C19*, siponimod/*CYP2C9*, irinotecan/*UGT1A1* abacavir/*HLA-B*57:01 and fluoropyrimidines/*DPYD*. *MTHFR* genotype is also analyzed following an institutional internal protocol, but no dose recommendation is done due to the lack of genotype-based clinical guidelines available. *RYR1* and *CACNA1S* genes are only analyzed in patients who have presented an event clinically compatible with malignant hyperthermia.

The majority of requests received can be classified into two groups: when a request is made for drugs for which the PGx testing is required before treatment prescription (preemptive genotyping in high-risk populations), petitions and samples are directly forwarded to the genetics department for sample processing and analysis. The final report with genetic data provided by the geneticist includes dose-adjustment recommendations provided by the clinical pharmacologist who takes into account the patient’s clinical background, individual interactions, and other factors is sent to the requesting service. When a medical case is referred where a PGx test could be deemed appropriate or a request is made for a drug without established clinical protocols, the Clinical Pharmacogenetics Unit’s clinical pharmacologist assesses and decides, by integrating available clinical information with up-to-date pharmacogenetic evidence, whether a PGx test is indicated. If the PGx test is recommended, the analysis is performed and a PGx report is generated. All PGx reports are performed according to the recommendations of the European Molecular Genetics Quality Network (EMQN) (European Molecular Genetics Quality Network, 2023). An example of our reports is shown in Supplementary Figure S1.

This study included patients with various pathologies with a PGx test performed in a local pharmacogenetic laboratory between August 2021 and September 2022. Demographic characteristics including age and gender as well as relevant future or current prescription, PGx data and diseases, were obtained from medical records and the laboratory information system over a 1-year period.

A descriptive analysis of the study population was carried out which included the number of tests performed, what gene was requested, genotyping results and its proportion attending the total amount of requests of each type. Variables were described using the number of participants (n), mean and it is minimum and maximum range. We then compared the results obtained during from 2014 to 2016 with those obtained from 2021 to 2022.

### 2.2 Pharmacogenetic testing methodology

Blood samples were obtained in Vacutainer EDTA tube (Becton Dickinson, Franklin Lakes, NJ, USA). DNA was isolated using a Chemagen extraction robot (Perkin-Elmer, Boston, MA, USA). Within our Hospital’s service portfolio, the technology used for each type of test was as follows: OpenArray^®^ technology (for the Pharmacogenetics of Fluoropyrimidines, Pharmacogenetics of Voriconazole, Pharmacogenetics of Thiopurines, Pharmacogenetics of Tacrolimus, Pharmacogenetics of Methotrexate, Pharmacogenetics of Siponimod); PCR combined with electrophoresis (for the Pharmacogenetics of Irinotecan); Real-time PCR (Hypersensitivity to Abacavir); Next-Generation Sequencing (Malignant Hyperthermia).

As previously described, most of the tests are carried out using the TaqMan™ OpenArray™ PGx Express Panel in the QuantStudio™ 12K Flex OpenArray^®^ System (Thermo Fisher Scientific, Waltham, MA, USA) following manufacturer instructions. This panel simultaneously analyses 120 single nucleotide variants (SNVs) in 8 genes using TaqMan™ probes. Each TaqMan™ SNV assay contains two allele-specific probes and a primer pair to detect the specific SNV target. The validation study conducted for this technology was previously published (Rosas-Alonso et al., 2021). For each PGx test available in our lab, predefined SNVs were analyzed (Supplementary Table S1). It is important to note that the OpenArray™ PGx Express Panel used in our laboratory is a predefined non-customizable assay and, of the total 120 SNVs included, our pharmacogenetics unit only analyzes routinely 27 SNVs from this panel. The rationale behind this selection is rooted in our commitment to evidence based medicine and adherence to international pharmacogenetic guidelines from groups such as the Association for Molecular Pathology (AMP), CPIC, DPWG and the SEFF. These 27 SNVs were carefully chosen based on the extensive literature and guidance provided by these authoritative sources.

The PCR reaction was conducted under the following conditions: an initial step of 10 min at 93°C, followed by 45 s at 95°C, 13 s at 94°C, and 2 min at 53.5°C for 50 cycles. Data acquisition and analysis were carried out utilizing the Thermo Fisher Cloud. Genotypes were determined through the utilization of a customized script and alleles were inferred according to drug-specific guidelines (Chandran et al., 2010; Tuková et al., 2010; Birdwell et al., 2015; Moriyama et al., 2017; Jin et al., 2018; Gonsalves et al., 2019; García et al., 2020).

Genotyping test for irinotecan pharmacogenetics study analyses by specific fluorophore-labeled PCR the most common genetic variant affecting *UGT1A1* gene, a TAn dinucleotide repeat variant (rs3064744) located in a TATAA consensus element in the *UGT1A1* promoter, The forward primer sequence was GAT​TTG​AGT​ATG​AAA​TTC​CAG​CCA​G, and the reverse primer sequence was CCA​GTG​GCT​GCC​ATC​CAC​T, which was fluorescently labeled with FAM. The PCR conditions consisted of an initial denaturation at 94°C for 3 min, followed by 31 cycles of denaturation at 94°C for 1 min, annealing at 60°C for 1 min, extension at 72°C for 7 min, and a final extension at 72°C for 7 min. Subsequently, fluorescent fragment sizing was performed using capillary gel electrophoresis. The analysis was carried out with GeneMapper^®^ Software v4.0. The star allele was determined based on the length of the amplicon.

The abacavir hypersensitivity study was conducted using genotyping test of *HLA-B* to establish presence or absence of *57:01 allele, associated hypersensitivity reactions to abacavir. The study was performed using the commercial GENVINSET^®^ HLA B57 kit (BDR, Zaragoza, Spain), which allows the detection of the HLA-B*57:01 allele using specific primers. The study was performed on the Cobas z 480 analyzer (Roche Diagnostics, Risch-Rotkreuz, Switzerland).

The malignant hyperthymia testing was performed by analyzing the coding regions of the *RYR1* and *CACNA1S* genes by a massive sequencing panel (NGS) of our own design using the KAPA HyperChoice technology (Roche Diagnostics, Risch-Rotkreuz, Switzerland). Sequencing was performed on the Novaseq6000 or HiSeq4000 platform (Illumina, San Diego, CA, USA). Bioinformatics analysis was carried out by the Bioinformatics Unit of the genetics department. Variant classification was performed based on the recommendations of the American College of Medical Genetics and Genomics (Richards et al., 2015) and variants were named according to the HGVS nomenclature (https://varnomen.hgvs.org/). The project accession PRJEB66347 in European Nucleotide Archive (ENA) has the high throughput sequencing data from cases of this study.

This study was conducted under the approval of the ethics committee of the La Paz University Hospital (PI-2915) and in conformance with the principles of the Declaration of Helsinki.

## 3 Results

### 3.1 Study population

**FIGURE 1:**
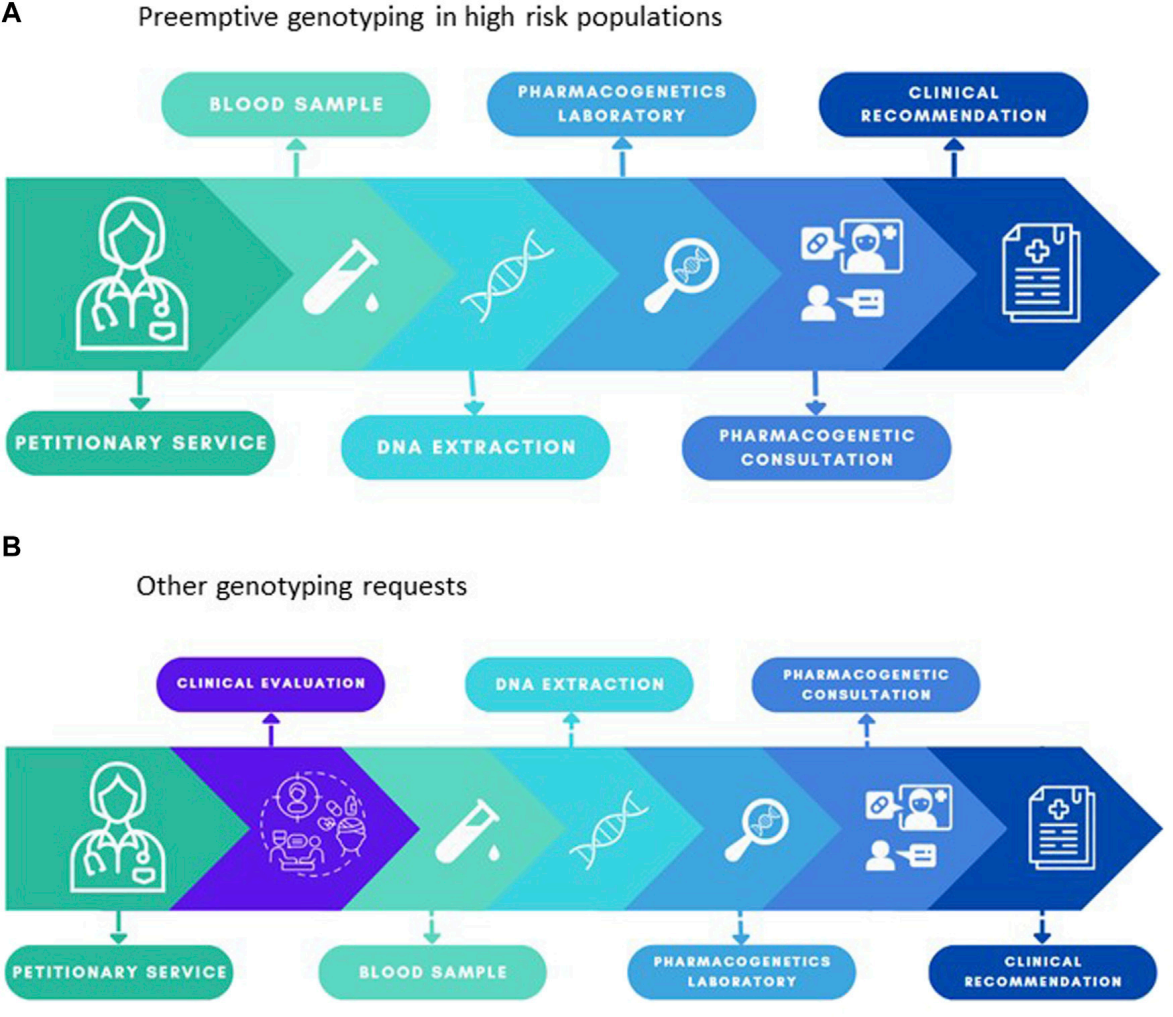
**(A)** Overall functioning of the Clinical Pharmacogenetics Unit for drugs for which the PGx testing is required before treatment prescription (preemptive genotyping in high-risk populations). **(B)** Overall functioning of the Clinical Pharmacogenetics Unit when a medical case is referred where a PGx test could be deemed appropriate or a request is made for a drug without established clinical protocols.

Since its establishment within LPUH in 2013, the Clinical Pharmacogenetics Unit has conducted genotyping for an approximate total of 5,000 patients. The overall hierarchical functioning of the Unit varies based on the type of request received and is summarized in Figures 1A, B. Activity has been uninterrupted save for a 4-month hiatus caused by the COVID-19 pandemic, when LPUH had to reallocate most of its financial and human resources to help mitigate the overwhelming demand in healthcare. The PGx tests requested and performed at our hospital between August 2021 and September 2022 numbered 1,145, involving 655 patients’ participants. A total of 655 patients were genotyped, total average age was 55.58 years (ranging from 0 to 94 years) with 43.7% (286 out of 655) being aged 65 or older. More than half of the sample subjects were male (55.8%). The LPUH is a public healthcare facility that caters to the inhabitants of the northern region of Madrid, where the demographic makeup is primarily Caucasian. Our Pharmacogenetics Unit receives referrals for various medical conditions, including cancer patients eligible for fluoropyrimidines and irinotecan treatments, transplant candidates undergoing tacrolimus therapy, oncology patients suitable for voriconazole treatment or those diagnosed with aspergillosis, individuals with immune-mediated dermatological, rheumatological, or digestive diseases who are potential tacrolimus candidates, patients considered for methotrexate therapy, and those with a prior adverse response to anesthetic agents or a family history of malignant hyperthermia.

### 3.2 Pharmacogenetic genotyping results

By comparison, the total number of PGx tests carried out between January 2014 and December 2016 (Borobia et al., 2018) by this same pharmacogenetic laboratory stood at 2,539 (approximately 846 tests per year). This indicates an average increase rate of 35% in testing activity over the course of approximately 6 years.

**FIGURE 2:**
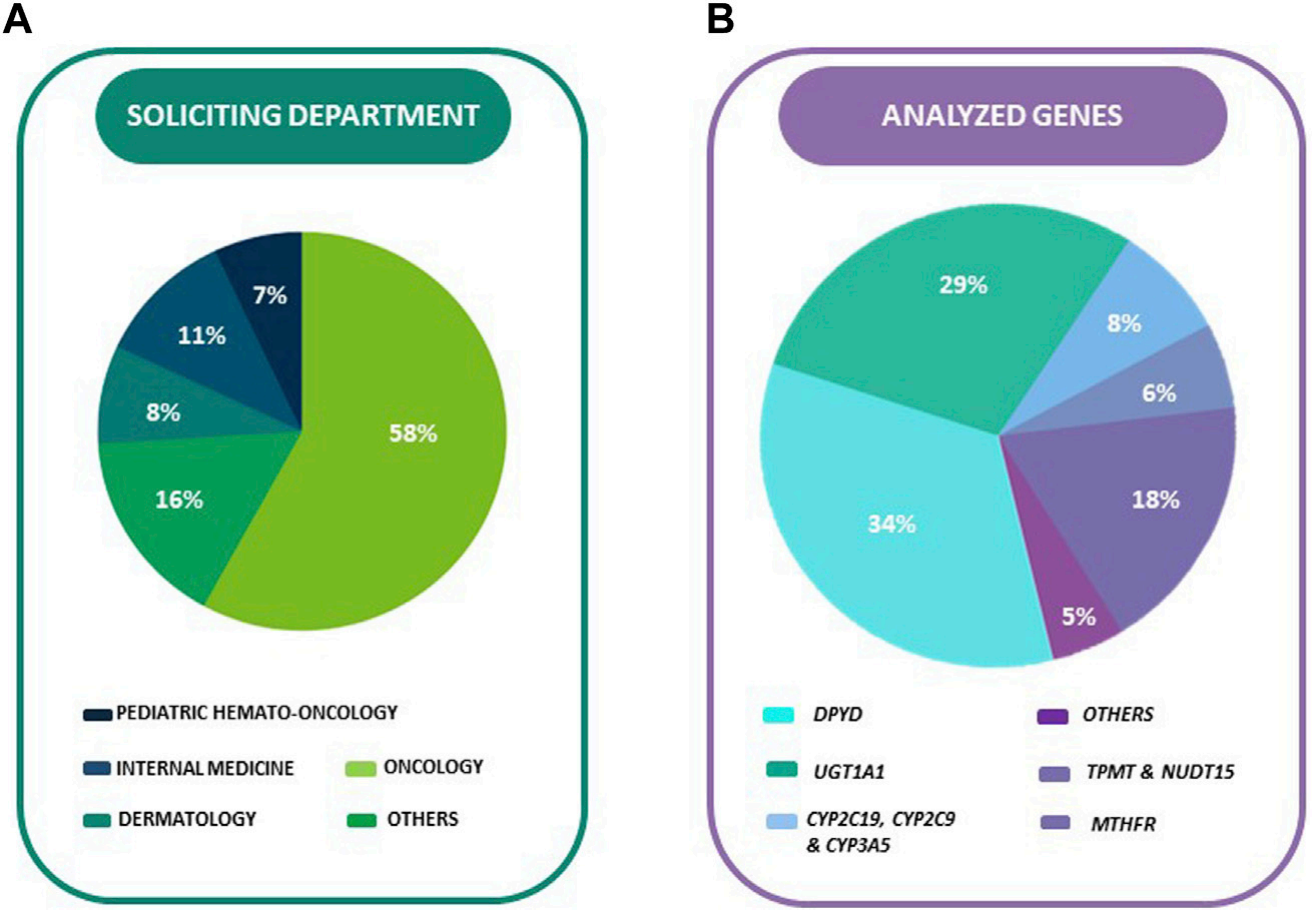
**(A)** Percentage (%) of analyzed genes in total sample. **(B)** Percentage (%) of analyzed genes in total sample.

Previously reported data revealed that the Internal Medicine/Infectious Disease department accounted for 76.3% (1939 out of 2,539) of the requested actionable genetic marker tests. However, in the current study, among the 17 different medical departments that sought PGx tests, the Oncology department accounted for the highest number, representing 58.47% (383 out of 655) of all genotyped patient (Figure 2A).

The department performing *DPYD* (95.36%, 370/388) and *UGT1A1* (92.55%, 311/336) genetic testing was limited almost exclusively to Oncology. Dermatology was the main department that requested PGx combined tests for *TPMT-NUDT15* (accounting for 29.41% of the requests, or 30 out of 102). Both Dermatology and Paediatric Haemato-Oncology had an equal number of requests for PGx tests for *MTHFR* (26.15% of the requests each, or 17 out of 65). Paediatric Haemato-Oncology had the highest percentage of requests for PGx tests for voriconazole (88.23% of their total requests, or 30 out of 34). Paediatric Nephrology was the main department ordering *CYP3A5* genotyping (46.15%, 18/39), while *CYP2C9* genotyping was exclusively ordered by the Neurology department (100%, 15/15). The HIV Clinic of Internal Medicine was the sole department ordering *HLA B57:01* genotyping.

Over a third of the 1,145 PGx tests (33.89%, 388/1,145 were performed for the genotyping of *DPYD* (Figure 2B), with the second most requested test being the genotyping of *UGT1A1* (29.34%, 336/1,145). The PGx tests performed for thiopurine S-methyltransferase (*TPMT*) and *NUDT15* gene accounted for 17.82% of the total (204/1,145), followed by *MTHFR* at 5.68% (65/1,145), abacavir *HLA B57:01* testing at 4.63% (53/1,145), *CYP3A5* at 3.41% (39/1,145), and Voriconazole PGx (*CYP2C19*) at 3.32% (38/1,145). The least requested tests were the ones involving *CYP2C9* at 1.22% (14/1,145) and malignant hyperthermia risk assessment, which accounted for only 0.70% of the total (4/1,145).

The mean response time (MRT) was determined as the number of elapsed days between the date of test request and the date of result report upload and exhibited a range of 1–176 days. The overall MRT for all tests was calculated to be 8.36 days, with a corresponding MRT for all PGx tests included in the OpenArray^®^ platform of 7.01 days. The MRT for *HLA B57:01* testing was found to be 15.75 days. The *RYR1* and *CACNA1S* genotyping tests exhibited an MRT of 91.2 days, this extended MRT is due to the complexity of the technique combined with limited financial resources and low incidence of clinical events compatible with malignant hyperthermia. Among the genes included in the OpenArray^®^, the mean response time (MRT) was consistently under 10 days across all instances. The shortest MRT was observed for the *DPYD* test (6.36 days), while the greatest MRT was seen for the *CYP3A5* test (8.66 days).

**FIGURE 3:**
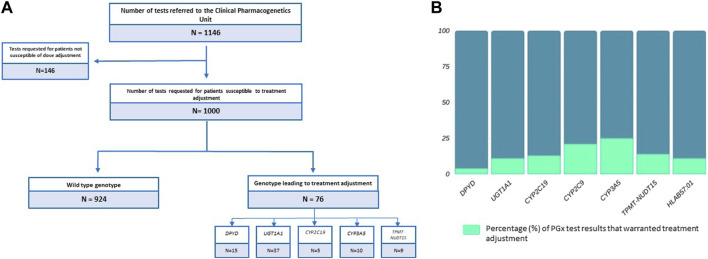
**(A)** Total number of pharmacogenetic tests performed in the study period. Number of tests requests requested for patients susceptible to treatment adjustment. Number of genotypes that led to treatment adjustment stratified by genes. **(B)** Percentage (%) of pharmacogenetic test results per gene that warranted a phenotype-based treatment adjustment.

Of the 655 individuals who underwent genetic testing, 83.21% (545/655) were performed prior to the initiation of treatment (preemptively). A total of 1,000 PGx tests were requested for individuals susceptible to receive a dose modification based on genotype. Among these cases, it was found that 7.60% (76 out of 1,000) of the individuals received a dose adjustment according to the genotype results (Figure 3A).

The results of the PGx testing of *DPYD*, *UGT1A1*, *CYP2C19*, *CYP3A5*, and *HLA-B57:01* indicated the following: 3.86% (15/388) of *DPYD* tests and 11.01% (37/336) of *UGT1A1* tests required treatment adjustments of 5-Fluorouracil/Capecitabine and Irinotecan, respectively. A proportion of 13.15% (5/38) of *CYP2C19* genetic tests prompted the administration of a different treatment from voriconazole. In the case of *CYP3A5*, 25.64% (10/39) of PGx tests led to adjustments in the starting dose of Tacrolimus in eligible kidney transplant patients. Finally, it was noted that 13.63% (9/66) of the *TPMT/NUDT15* tests conducted resulted in a modification of the dose of either Azathioprine or 6-Mercaptopurine. Only 3 out of 14 *CYP2C9* tests warranted dose modification of siponimod. In addition to pharmacogenomic tests performed using OpenArray technology, the results of *HLA-B57:01* genotyping indicated that 11.32% (6/53) of patients were at elevated risk for developing abacavir hypersensitivity, thereby requiring an alternative Highly Active Antiretroviral Therapy (HAART) regimen (Figure 3B).

With respect to the *RYR1* gene, a significant proportion (50%, 2/4) of individuals possessed at least one clinically relevant variant in the *RYR1* gene. It is worth noting that all genetic assessments for malignant hyperthermia were performed on patients who had previously experienced a compatible adverse event in the operating room, hence none of these tests were conducted preemptively. One of the identified variants, NM_000540.2 (*RYR1*):c.15014C>T(p.Thr5005Met), was classified as a variant of uncertain significance, which indicates that further analysis, such as a more comprehensive assessment, would be required to draw definitive conclusions regarding the subject’s increased or decreased risk of developing malignant hyperthermia. On the other hand, the other detected variant, NM_000540.2 (*RYR1*):c.14545G>A p.(Val4849Ile), was classified as a pathogenic variant.

Finally, Table 1 showcases the absolute number of results obtained for each different genotype as well the proportion observed.

**TABLE 1:** Results obtained for each different genotype and observed proportion.

Gene phenotype	Absolute (n)	Observed percentage
*DPYD* 2	373	96.13%
*DPYD* 1.5	12	3.09%
*DPYD* 1	3	0.77%
*UGT1A1* *1/*1	162	48.21%
*UGT1A1* *1/*28	136	40.48%
*UGT1A1* *28/*28	36	10.71%
*UGT1A1* 1*/*37	1	0.30%
*UGT1A1* *28/*37	1	0.30%
*CYP2C19* *1/*1	23	60.53%
*CYP2C19* *1/*2	7	18.42%
*CYP2C19* *2/*3	1	2.63%
*CYP2C19* *1/*17	3	7.89%
*CYP2C19* *2/*17	1	2.63%
*CYP2C19* *1/*8	1	2.63%
*CYP2C19* *17/*17	2	5.26%
*CYP2C9* *1/*1	6	42.86%
*CYP2C*9 *1/*2	5	35.71%
*CYP2C9* *1/*3	1	7.14%
*CYP2C9* *2/*3	2	14.29%
*CYP3A5* *1/*1	1	2.56%
*CYP3A5* *1/*3	9	23.08%
*CYP3A5* *3/*3	28	71.79%
*CYP3A5* *3/*7	1	2.56%
*MTHFR* GG	26	40.00%
*MTHFR* GA	26	40.00%
*MTHFR* AA	13	20.00%
*TPMT* *1/*1	95	93.14%
*TPMT* *1/*3A o *3B/*3C	7	6.86%
*NUDT15* *1/*1	100	98.04%
*NUDT15* *1/*3 (*1/*2)	2	1.96%
*HLA B57:01* NEGATIVE	47	88.68%
*HLA B57:01* POSITIVE	6	11.32%
*RYR1* PATHOLOGICAL	2	50.00%
*RYR1* NON-PATHOLOGICAL	2	50.00%
*CACN1S* PATHOLOGICAL	0	0.00%
*CACN1S* NON-PATHOLOGICAL	4	100.00%
TOTAL PGX TEST	1,145	

## 4 Discussion

The role of preemptive pharmacogenetics in enhancing treatment efficacy and significantly reducing adverse drug reactions is well established, as evidenced by recent publications (Turongkaravee et al., 2021; Swen et al., 2023). We have conducted a comprehensive analysis of the performance of our Clinical Pharmacogenetic Unit by examining the activities performed over a 1-year period. This study aims to evaluate the trends and outcomes observed in both test petitions and results, as well as the challenges faced by our Clinical Pharmacogenetics Unit. In order to showcase the advancements made, we performed a head-to-head comparison of real-world data with previously published activity results from the same Unit, over two different time periods. By comparing and contrasting our findings with previous research performed within the same setting, we seek to provide insights into significant achievements, identify areas for improvement, and outline future directions that have the potential to advance and enhance precision medicine practices.

Our analysis encompassed a total of 1,000 PGx tests specifically requested for individuals deemed susceptible to benefit from a dosage/treatment modification based on their genetic profile. The results revealed that a noticeable amount of these tests, 7.60% (76/1,000), led to modified treatments being implemented according to the genotype outcome. Few evidence has been published describing global prevalence of PGx variants in a similar population cohort to contrast this figure (Runcharoen et al., 2021).

Despite currently being the two most commonly requested PGx tests, fluoropyrimidines and immunosuppressants were found to constitute only 2.7% and 6.7%, respectively, of our previously reported overall laboratory activity (Borobia et al., 2018). This further reflects the increase of activity of both our laboratory and oncology services something concurrent with other reported results within Europe (Bignucolo et al., 2023). However, our proportion of *DPYD* of patients with decreased allele activity score (3.86%) differed from similar studies conducted in European cohorts that reported a higher prevalence, albeit in a larger sample size (Pallet et al., 2020) as well as the estimates provided by the European Medicines Agency (EMA) (European Medicines Agency, 2020). No definitive explanation for these differences was discerned; however, it is plausible that variations in demographics, ethnic diversity, and sample size may have contributed to the observed disparities.

In contrast, our *CYP2C19* and *CYP3A5* data reveals a higher variant prevalence compared to the figures described in Caucasian and European populations (Hicks et al., 2017; Buendía et al., 2020). Thus, the genetic characteristics observed in our region could potentially influence the prevalence rates observed in genetic studies. These results provide compelling support for the implementation of preemptive testing strategies of both these genes and highlight the heterogeneity in genotypes between different population subsets within Europe. In relative terms, our results show that the highest proportion of treatment adjustments was observed for tacrolimus among eligible kidney transplant patients, with 25.64% of PGx tests leading to modifications in the starting dose. This figure aligns more closely with the results published in Eastern populations, suggesting a potential shift in the demographic of the treated population (Maurya et al., 2022).

In absolute terms, *DPYD* and *UGT1A1* testing led to the most significant treatment adjustments, identifying a total of 15 and 37 patients respectively that required a modified treatment approach. Both results emphasize the clinical value of incorporating PGx testing into routine practice and shed light on the potential benefits of personalized medicine in improving patient care.

The comparison of our data with a similar study conducted in a different setting and over a more extended timeframe is of particular significance. Zhang *et al.*, despite exploring a lesser number of genotypes (*CYP2C19*, *CYP2C9*, *MTHFR*, *VKORC1* and *ALDH*) highlighted *P450 2C19* as their most frequently requested test, constituting 50.2% of their total inquiries (Zhang et al., 2021). In contrast, our study found that this specific test accounted for a mere 3.31% of the overall requested PGx tests. Notably, Zhang *et al.* reported that a majority of PGx requests in their setting originated from the Cardiology and Critical Care Units, representing a combined 55% of the total requests (Zhang et al., 2021). Surprisingly, the Oncology Department contributed only 0.6% of the total PGx requests in their study. However, the authors did not provide details on the availability of *DPYD* or *UGT1A1* testing, which could potentially explain these substantial variations (Zhang et al., 2021). While it is crucial to recognize that these differences could be influenced by the indications specified by the Chinese Regulatory Authority, it is important to note that no information pertaining to this aspect was found in our research.

Previously published data by Borobia *et al.* analyzed the activity of LPUH PGx testing laboratory. The study spanned 3 years from January 2014 to December 2016. During this period, the laboratory conducted 2,539 PGx test (Borobia et al., 2018), 2,287 excluding *IL28B* genotyping which is no longer available due to the advent of new retroviral therapies (Ghany et al., 2020). When compared to our current data, testing activity has increased roughly 35% over the course of nearly 6 years. While our test menu has changed to accommodate emerging pharmacogenetic insights, the key driver behind the increased demand for testing might have been the regulatory guidance and heightened awareness among healthcare providers.

When examining specific test requests, significant disparities emerge upon comparing our data with that presented in the aforementioned paper. Particularly striking is the discernible decline observed in *HLA-B57:01* screening requests (previously reported to be the most solicited test) which has diminished by nearly 91% annually (corresponding to a net disparity of 506 petitions) when compared to our present findings. This unmistakably signifies a change in the management of HIV patients by healthcare professionals, wherein the utilization of abacavir has been relegated to a later stage when compared to the contemporary approach of HAART combinations (Ruiz-Algueró et al., 2022).

Of the remaining available tests, the second most petitioned PGx test reported by Borobia *et al.*, was *TPMT*, which contrasts greatly with our data. In our study, the medical oncology department emerged as the primary requester of PGx tests, primarily due to the high volume of patients attended with colorectal cancer. This data is consistent with both the mean age of all patients genotyped, as well as the two most petitioned tests. This could be explained, at least in part, by the inclusion of *DPYD* genotyping for these patients in the summary of product characteristics (SmPC) of capecitabine and 5-FU and security alerts issued by the Spanish Agency of Medicines and Medical Devices (Agencia Española del Medicamento y Productos Sanitarios, 2020), despite having proven to be a safe, effective, and cost-effective years prior (Henricks et al., 2018). This correlation has been reported by other laboratories across Europe (Bignucolo et al., 2023). Similarly, the genotyping of *UGT1A1* is indicated prior to the administration of irinotecan (European Medicines Agency, 2021). Given the significant impact of colorectal cancer and the therapeutic implications of *DPYD* and *UGT1A1* genotyping, it is understandable that medical oncology had the highest demand for PGx testing services in this study. This increase of activity showcases the impact Regulatory Agencies in enabling the widespread acceptance of PGx testing by encouraging the implementation of preemptive genotyping strategies.

These findings, as well as the increase in *DPYD* and *UGT1A1* PGx petitions, underscore the clinical relevance and practical application of genotype-guided medication management in optimizing treatment efficacy and patient outcomes. They also showcase the impact of incorporating preemptive genotyping strategies into clinical guidelines and SmPC’s in enabling the implementation of PGx testing.

Despite the promising findings, we continue to encounter difficulties that exemplify why the widespread implementation of PGx testing remains a challenge. One such obstacle is the interpretation of a test result performed at a different laboratory (with a possibly different technique), something not uncommon given the increasing flux of patients across borders.

Another significant issue we encountered pertains to the lack of expertise and awareness by the petitioner, which occasionally led prescribing clinicians to order the same test repeatedly (duplicated tests were excluded from our analyses) or to do so in cases that were incongruous: a petition for a PGx test for *TPMT* in a patient that has been subjected to an orthotopic liver transplant, where the donor’s genotype can condition whether or not the patients suffer side effects in response to treatment (Breen et al., 2005).

Likewise, it was frequently observed that petitioners would request a PGx test, such as *CYP3A5*, for a kidney transplant recipient who was already undergoing treatment adjustment as per trough blood concentrations. In such a scenario, patient genotype would be of little clinical relevance, with blood concentrations guiding any treatment modifications (Borobia et al., 2009).

In our hospital, we have developed an internal record-keeping system that helps us identify instances of duplicate orders or cases where pharmacogenetic testing has been previously conducted for a particular patient. This internal system serves as a safeguard to prevent unnecessary repetition of tests. Additionally, for patients already undergoing therapeutic drug monitoring, such cases are identified through a manual review of the patient’s medical history by the clinician responsible for handling the test request. This review process also allows us to prioritize and manage cases that require urgent pharmacogenetic results.

It therefore becomes apparent that genetic information is complex, and effectively translating it into actionable recommendations for clinicians requires clear guidelines and standardized reporting formats. Our Clinical Pharmacogenetics Unit exemplifies a model useful to bridge some of these challenges by integrating the interpretation of complex genetic information with the patient’s clinical status, therapeutic goals, and concomitant therapy. Something only possible by establishing a multidisciplinary unit that comprises expert geneticists and clinical pharmacologists with pharmacogenetic expertise.

The findings of this study should be interpreted within the context of several limitations. Despite being active for 10 years, the data collected by the Clinical Pharmacogenetics Unit has been subject to various information system changes as the LPUH adapted its Information Technology infrastructure, and electronical medical health records platform among others. This precluded the study from spanning a longer time period, as collected data from the inception of the Unit was limited. The impact of the COVID-19 pandemic also hindered the activity of the Unit, not only because it suffered a complete cessation of activity during the lockdown period, but because in order to ease the economic burden caused by the pandemic financial reallocations were carried throughout the SNHS that rippled through units and departments that, despite being of great relevance for individual patient care, were not considered a priority at the time (Carrera-Hueso et al., 2021). The absence of robust cost-effective studies that warrant public health expenditure in PGx strategies such as the ones carried out by our Unit further worsened this issue. In this sense, promising new studies are currently underway, with some being developed within our own research group. These studies may offer compelling arguments regarding the impact of pharmacogenetics on SNHS sustainability (Monserrat Villatoro et al., 2020).

This study represents a significant contribution as it is, to the best of our knowledge, the first to offer comprehensive insights into the activity of a Clinical Pharmacogenetics Unit over two different time periods in the same setting. By doing so, we provide a deeper understanding of PGx practices in a real-world setting, with both their challenges and achievements. The findings presented highlight a notable rise in the utilization of PGx testing, indicating a growing awareness and acknowledgment among healthcare professionals regarding its significant role in customizing treatments according to individual genetic profiles. This increasing acceptance signals a shift towards a more personalized medicine, emphasizing the aim of delivering optimized and safe therapeutic interventions for every patient. By leveraging the insights provided by PGx testing, healthcare providers can make informed decisions and ensure the administration of treatments that are both efficacious and tailored to each patient’s unique genetic makeup.

The real-world data presented in this study contributes to the growing body of evidence supporting the integration of PGx testing into daily clinical practice, allowing for tailored treatments based on individual genetic profiles, ultimately enhancing patient care. The implementation of PGx strategies that include a multidisciplinary approach that includes a geneticist and a clinical pharmacologist has the potential to significantly enhance the efficient utilization of available resources within healthcare systems, bringing us one step closer to a truly individualized healthcare. By optimizing the efficacy of current treatments and reducing the incidence of adverse events, pharmacogenetics can also contribute to a more sustainable healthcare system. However, further studies are needed to comprehensively explore and validate the full extent of these benefits, ensuring that the integration of pharmacogenetic and economic approaches is evidence-based and widely applicable.

## Data Availability

The datasets presented in this study can be found in online repositories. The names of the repository/repositories and accession number(s) can be found below: European Nucleotide Archive (ENA), PRJEB66347.
