# Supplementary material for: Advancing pharmacogenetic testing in a tertiary hospital: a retrospective analysis after 10 years of activity
Source: Front Pharmacol. 2023 Oct 19;14:1292416. doi: 10.3389/fphar.2023.1292416 (PMC10622662; doi:10.3389/fphar.2023.1292416)
Supplement: Supplementary file 1 [file Table1.docx]

| Pharmacogenetic Test | Gene | Predefined SNV | Corresponding Allele (*) |
| --- | --- | --- | --- |
| Pharmacogenetics of Fluoropyrimidines | *DPYD* | rs3918290  rs55886062  rs67376798  rs56038477  rs115232898 | *2A *13 - HapB3 - |
| Pharmacogenetics of Voriconazole | *CYP2C19* | rs4244285  rs4986893  rs28399504  rs56337013  rs72552267  rs72558186  rs41291556  rs17884712  rs6413438  rs12248560  rs12769205 | *2 *3 *4 *5 *6 *7 *8 *9  *10 *17 *2 & *35 |
| Pharmacogenetics of Thiopurines | *TPMT* | rs1800462  rs1800460  rs1142345  rs1800584 | *2 *3A/*3B *3A/*3C *4 |
|  | *NUDT15* | rs116855232 | *2 & *3 |
| Pharmacogenetics of Tacrolimus | *CYP3A5* | rs776746  rs10264272  rs41303343 | *3 *6 *7 |
| Pharmacogenetics of Methotrexate | *MTHFR* | rs1801133 | - |
| Pharmacogenetics of Siponimod | *CYP2C9* | rs1799853  rs1057910 | *2 *3 |

Supplementary Table 1: Pharmacogenetic drug tests performed during routine clinical practice at the LPUH Clinical Pharmacogenetics Unit, their predefined genes,single nucleotide variants (SNVs) and corresponding alleles.
