# Supplementary material for: Advancing pharmacogenetic testing in a tertiary hospital: a retrospective analysis after 10 years of activity
Source: Front Pharmacol. 2023 Oct 19;14:1292416. doi: 10.3389/fphar.2023.1292416 (PMC10622662; doi:10.3389/fphar.2023.1292416)
Supplement: Supplementary file 2 [file DataSheet1.PDF]

PHARMACOGENETICS UNIT<br><br>Address and telephone	**First name:**<br>**Surname:**<br>**Sex:**<br>**Date of birth:**<br>**Date of sample recieved:**<br>**Report date:**

PHARMACOGENETIC REPORT

Reason for request / clinical indication: planned cancer treatment with fluoropyrimidines
Test requested: Pharmacogenetic testing
Reference department: Oncology Department
Reference clinician:
Report to:

Sample Type:
Sample batch ID:

TEST RESULTS:

DPYD: Intermediate metabolizer (DPD activity score = 1) (*1/*2A)

This result indicates that the patient has one copy of a normal function allele (*1) and one copy of a no function allele (*2A) of the *DPYD* gene. Based on the genotype result this patient is predicted to be a ***DPYD* Intermediate Metabolizer** with a **DPD activity score of 1**. This patient may be **at risk for adverse drug reactions** to medications that are metabolized by DPD (i.e. 5-fluorouracil, capecitabine, and tegafur) and therefore a **dose adjustment may be required.**

Gene (RefSeq)	rs ID (Allele)	Variant (cDNA)	Result	Diplotype
DPYD (NM_000110.4)	rs3918290 (*2A)	c. 1905+1G>A	**GA**	***1/*2A**
DPYD (NM_000110.4)	rs55886062 (*13)	c.1679T>G	TT	
DPYD (NM_000110.4)	rs67376798 (not available)	c.2846A>T	AA	
DPYD (NM_000110.4)	rs56038477 (not available)	c.1236G>A	GG	

INTERPRETATION:

Fluropyrimidines are metabolized by the *DPYD* gene. *DPYD* gene encodes the enzyme dihydropyrimidine dehydrogenase (DPD). This enzyme is involved in the degradation pathway of **fluoropyrimidines**, such as 5-fluorouracil, capecitabine, and tegafur. Patients treated with fluoropyrimidines who have complete or partial deficiency of DPD activity are at increased risk of adverse reactions when treated with these drugs. The European Medicines Agency recommends the study of the following variants associated with fluoropyrimidenes toxicity: c.1905+1G>A, c.1679T>G, c.2846A>T and [c.1236G>A; c.1129-5923C>G] (HapB3). The variants c.1905+1G>A, c.1679T>G are considered non-functional variants while the variants c.2846A>T and [c.1236G>A; c.1129-5923C>G] (HapB3) have decreased function. No recommendations are provided for other variants in *DPYD* or other genes (PMID: 31745289). The Clinical Pharmacogenetics Implementation Consortium (CPIC) released guidelines for fluoropyrimidine treatment depending on DPYD activity score, indicating that patients who are heterozygous (intermediate activity) should reduce starting dose by 25% to 50% (PMID: 29152729).

RECOMMENDATIONS

-The patient has a fluoropyrimidine intermediate metabolizer phenotype. Patients with DPYD activity of 1 require a starting **dose reduction of 50%** followed by dose titration based on toxicity or pharmacokinetics.

METHODOLOGY AND LIMITATIONS

- **DNA isolation:** Chemgene
- ***DPYD* Genotyping Study:** analysis of single nucleotide polymorphism (SNP) using OpenArray™ technology. The analysis of the results is carried out with Thermo Fisher Cloud.
- **SNPs analysed**: rs3918290, rs55886062, rs67376798 and rs56038477 of the *DPYD* gene
- **Analytical sensitivity:** ≥87.5% (PMID: 34116532).

Limitations:

- The DPD activity score of 2 is assigned due to the absence of variants in the analysed rsIDs. It is always possible that a new, previously undiscovered (and therefore un-interrogated) site of variation may confer loss-of-function in an individual, and thus lead to the rare possibility of a non-functional allele being erroneously called as DPD activity score of 2.
- These techniques do not allow the detection of copy number alterations of the analysed gene.
- These techniques do not difference whether the polymorphisms are in *cis* or *trans*.
- HapB3 consists of three intronic variants (c.483+18G>A - rs56276561, c.680+139G>A - rs6668296 and c.959-51T>C - rs115349832) and one synonymous variant (c.1236G>A - rs56038477) that are in linkage disequilibrium with a deep intronic variant (c.1129-5923C>G; rs75017182). In this study only the c.1236G>A variant is analysed.

Dr. XX
Molecular Geneticist

Dr. XX
Clinical pharmacologist
